# Isoeucommin A attenuates kidney injury in diabetic nephropathy through the Nrf2/HO‐1 pathway

**DOI:** 10.1002/2211-5463.13251

**Published:** 2021-07-24

**Authors:** Qi Huang, Dong‐Sheng Ouyang, Qiong Liu

**Affiliations:** ^1^ Department of Clinical Pharmacology Xiangya Hospital Central South University Changsha China; ^2^ Institute of Clinical Pharmacology Hunan Key Laboratory of Pharmacogenetics Central South University Changsha China; ^3^ Department of Pharmacy Xiangya Hospital Central South University Changsha China; ^4^ National Clinical Research Center for Geriatric Disorders Changsha China; ^5^ Hunan Key Laboratory for Bioanalysis of Complex Matrix Samples Changsha Duxact Biotech Co., Ltd. China; ^6^ Department of Oncology Xiangya Hospital Central South University Changsha China

**Keywords:** diabetic nephropathy, human renal mesangial cells, Isoeucommin A, Nrf2/HO‐1, oxidative stress

## Abstract

Diabetic nephropathy (DN) is a common complication in patients with diabetes and a leading cause of mortality. The management of DN in the clinic still remains a challenge. Therefore, the identification of novel compounds for DN treatment and their characterization in preclinical DN models are crucial. Isoeucommin A is a lignan compound isolated from *Eucommia ulmoides* Oliv, which has not been studied in detail. Our aim was to investigate the effect of Isoeucommin A in DN and to elucidate the molecular mechanisms though which Isoeucommin A acts *in vitro* and *in vivo*. We first isolated and purified Isoeucommin A by microporous resin column chromatography and studied the mass spectrogram, as well as the structure of Isoeucommin A, by high‐resolution electrospray ionization mass spectroscopy and NMR, respectively. We further established an *in vivo* rat DN model and measured the changes of blood glucose, body weight, kidney index (KI), blood urea nitrogen, creatinine (CRE), glutathione, malondialdehyde (MDA), SOD, albumin (ALB) and urinary ALB to CRE ratios on treatment with Isoeucommin A. In addition, we measured SOD, MDA, glycogen synthase kinase‐3β (GSK‐3β), phosphorylated (p)‐GSK‐3β, nuclear factor erythroid‐derived 2‐related factor 2 (Nrf2) and heme oxygenase‐1 (HO‐1) levels by quantitative real‐time PCR and western blot, and estimated cell viability by a 3‐(4,5‐dimethylthiazol‐2‐yl)‐2,5‐diphenyl‐tetrazolium bromide assay. After Isoeucommin A treatment, body weight, as well as SOD, glutathione, HO‐1 and Nrf2 expression levels, in DN rats increased in a dose‐dependent manner. In contrast, the levels of blood glucose, KI, blood urea nitrogen, CRE, urinary ALB to CRE ratio, tumor necrosis factor‐α, interleukin‐1β, interleukin‐6 and MDA decreased significantly. In addition, Isoeucommin A protected H_2_O_2_‐stimulated renal tubular epithelial cells from oxidative stress and activated the Nrf2/HO‐1 signaling pathway in high‐glucose‐stimulated human renal mesangial cells. In conclusion, Isoeucommin A could alleviate inflammation and oxidative stress in *in vitro* and *in vivo* DN models and thus attenuate kidney injury by activating the Nrf2/HO‐1 signaling pathway. Isoeucommin A could have the potential to be used as an effective drug for the treatment of DN.

## Graphical abstract

Diabetic nephropathy (DN) is a common complication in patients with diabetes. Here we investigated Isoeucommin A treatment *in vitro*, as well as in a rat model of DN. We observed a dose‐dependent increase in the expression levels of SOD, glutathione, nuclear factor erythroid‐derived 2‐related factor 2, heme oxygenase‐1 and p‐GSK‐3β/GSK‐3β in DN rats upon treatment. In contrast, tumor necrosis factor‐α, interleukin‐1β, interleukin‐6 and malondialdehyde levels decreased significantly. Isoeucommin A protected H_2_O_2_‐stimulated renal tubular epithelial cells from oxidative stress and activated the nuclear factor erythroid‐derived 2‐related factor 2/heme oxygenase‐1 signaling pathway in high‐glucose‐stimulated human renal mesangial cells.

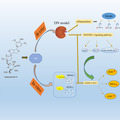

**Abbreviations**
ALB: albumin
AR: aldose reductase
BG: blood glucose
BUN: blood urea nitrogen
CRE: creatinine
DMEM: Dulbecco’s modified Eagle’s medium
DN: diabetic nephropathy
EUO: *Eucommia ulmoides* Oliv
GSH: glutathione
HO‐1: heme oxygenase‐1
HRESIMS: high‐resolution electrospray ionization mass spectroscopy
HRMC: human renal mesangial cell
IHC: immunohistochemistry
IL‐1β: interleukin‐1β
IOD: integral optical density
KI: kidney index
MDA: malondialdehyde
MTT: 3‐(4,5‐dimethylthiazol‐2‐yl)‐2,5‐diphenyl‐tetrazolium bromide
Nrf2: nuclear factor erythroid‐derived 2‐related factor 2
RTEC: renal tubular epithelial cell
STZ: streptozotocin
TNF‐α: tumor necrosis factor‐α
UACR: urinary ALB to CRE ratio

Diabetic nephropathy (DN) is a life‐threatening chronic kidney disease caused by hyperglycemia, leading to hypertension, glomerular inflammation, anemia and many other severe symptoms [1]. In recent years, the incidence of DN has increased gradually [2]. The polyol pathway and its rate‐limiting enzyme aldose reductase (AR) have been proved to play essential roles in DN. The increased polyol pathway flux can lead to oxidative stress, nonenzymatic protein glycosylation and activation of protein kinase C [3]. Among them, AR plays an important pivotal role, and the inhibition of AR has become a popular target in the research and development of DN‐related prevention and treatment drugs. AR inhibitors are mainly chemical drugs that include hydantoins, carboxylic acids and heterocyclic compounds. However, many of them have some drawbacks, such as low selectivity and large clinical dosage [4]. Therefore, it is of great significance to find new AR inhibitors with high economic value, high efficiency and lower toxicity.

*Eucommia ulmoides* Oliv (EUO) contains various bioactive chemicals, especially lignans, which possess potent antioxidant, anti‐inflammatory and anti‐AR activities [5, 6, 7]. It was reported that EUO could ameliorate glucotoxicity by suppressing advanced glycation end products in diabetic mouse kidneys [8]. The extract of EUO has a good effect on DN, as well as type 2 diabetes [9, 10]. In our previous studies, we separated and purified lignans and phenolic constituents from EUO [11]. In this research, we identified Isoeucommin A, a little‐studied lignan compound isolated from EUO. Our initial experiments suggested that Isoeucommin A has an anti‐inflammatory effect on human mesangial cells cultured in high glucose, which indicated that Isoeucommin A might have therapeutic effects on DN. In this study, we have explored the potential role of Isoeucommin A on DN.

Nuclear factor erythroid‐derived 2‐related factor 2 (Nrf2) is one of the critical cellular defense factors to counteract oxidative stress [12]. Heme oxygenase‐1 (HO‐1), an important target gene of Nrf2, is considered a protective gene in the kidney. It participates in the degradation of oxidized heme and produces anti‐inflammatory, antioxidant and antiapoptotic products [13]. Many studies have demonstrated that activation of the Nrf2/HO‐1 pathway can effectively prevent renal injury in DN mice and improve inflammatory response and oxidative stress [14, 15, 16]. Recent research has indicated that lignans can protect hippocampal neuronal cell line HT22 from oxidative stress by up‐regulating the Nrf2/HO‐1 signaling pathway [17]. We found that Isoeucommin A can regulate Nrf2 in our previous experiments. However, whether Isoeucommin A could play a part in kidney injury in DN through Nrf2/HO‐1 has not been elucidated. Herein, a DN rat model was established by intraperitoneal injection of streptozotocin (STZ) and human renal mesangial cells (HRMCs). The effect of Isoeucommin A on H_2_O_2_‐induced renal tubular epithelial cells (RTECs) was investigated. We have determined tumor necrosis factor‐α (TNF‐α), interleukin‐1 (IL‐1), IL‐6, SOD, malondialdehyde (MDA) and glutathione (GSH) levels to characterize the effect of Isoeucommin A on inflammatory response and oxidative stress response in DN rats. This work has provided a potential economic and efficient drug for the clinical treatment of DN.

## Materials and methods

### Animal model

Forty Sprague Dawley male rats (8 weeks old) that were raised in the animal facility center of Xiangya Hospital were purchased from Hunan SJA Laboratory Animal Co., Ltd (Changsha, China). All experimental protocols were approved by the Institutional Animal Ethics Committee at Central South University. The ethics approval number was provided (Identification Number: 2019030143) by the ethics committee. Rats were randomly divided into the control group, DN model group, Isoeucommin A low‐dose group (Low‐Isoeucommin A), Isoeucommin A medium‐dose group (Medium‐Isoeucommin A) and Isoeucommin A high‐dose group (High‐Isoeucommin A). The STZ‐DN rat model was established as described by the previous study [18]. The rats in the DN and Isoeucommin A groups were treated with 60 mg·kg^−1^ STZ (V900890‐1G; Sigma, St. Louis, MO, USA) intraperitoneally. After 4 weeks, the rats in high‐Isoeucommin A, medium‐Isoeucommin A and low‐Isoeucommin A dose groups were administered via lateral tail vein injection at 10, 5 and 2.5 mg·kg^−1^·day^−1^, respectively. At the same time, the rats in the DN and control groups were given the same amount of physiological saline (1 mL·100 g^−1^·day^−1^) by gavage. All rats were given gavage for 28 days continuously. The blood glucose (BG) and body weight of rats in each group were monitored weekly (days 0, 7, 14, 21 and 28). After 8 weeks of drug intervention, urine was collected in a metabolic cage for 24 h. The next day, after the fasting rat tail vein blood was taken, the animals were sacrificed.

### Extraction and separation of Isoeucommin A

The water reflux extraction method was used to extract 10 kg of EUO bark twice; then the aqueous solution was concentrated under vacuum conditions to obtain the extract. The extracted parts were separated and purified by macroporous resin column chromatography. The macroporous resin HPD‐100 was gradient eluted with 100% to 5% ethanol to obtain 70% ethanol eluent. The 70% ethanol eluent was eluted by silica gel column chromatography with the volume ratio of dichloromethane (DCM ): MeOH (100 : 0 to 50 : 50), and the eluents with the volume ratio of DCM : MeOH (100 : 15 to 100 : 20) were collected. The obtained eluent was separated and purified on a semipreparative HPLC column with a mobile phase of 22–28% acetonitrile–water solution. The product was obtained after collecting the peak time of about 13.31 min.

### Mass spectrometry analysis of Isoeucommin A

After separation and purification, high‐resolution electrospray ionization mass spectroscopy (HRESIMS) was measured using an Agilent 6545A Q/TOF mass spectrometer (Agilent Corp., Santa Clara, CA, USA). The operating parameters were as follows: drying N_2_ gas flow rate, 6.8 L·min^−1^; temperature, 325 °C; nebulizer, 35 Psig (pounds per square inch gauge); capillary, 4000 V; fragment voltage, 110 V. The samples were analyzed in positive ion mode and mass spectra data recorded across an *m/z* range of 50–1000. Reference masses of 121.0509 (purine) and 922.0098 (HP‐0921) were used for internal mass calibration during runs in positive ion mode.

### NMR

The Isoeucommin A structure was obtained from NMR carbon spectrum data [δ unit: parts per million (PPM)]. The data are shown in Table 1.

**Table 1:** The NMR carbon spectrum data of Isoeucommin A.

Position	δH (J in Hz)	δC	Position	δH (J in Hz)	δC
1	–	131.7	6′	6.93 (1H, dd, 8.4, 1.8)	118.4
2/6	6.81 (1H, d, 1.8)	103.1	7′	4.73 (1H, d, 4.8)	85.7
3/5	–	147.9	8′	3.15 (1H, m, overlap)	54.1
4	–	136.1	9′	4.27 (1H, m, overlap) 3.89 (1H, m, overlap)	71.3
7	4.78 (1H, d, 4.2)	86.2	3/5‐OCH3	3.86 (6H, s, overlap)	55.4
8	3.15 (1H, m, overlap)	54.1	3′‐OCH3	3.88 (6H, s, overlap)	55.4
9	4.27 (1H, m, overlap) 3.89 (1H, m, overlap)	71.4	1″	–	101.4
1′	–	134.8	2″	3.50 (1H, m)	73.5
2′	7.05 (1H, d, 1.8)	110.2	3″	3.48 (1H, m)	76.4
3′	–	149.6	4″	3.42 (1H, m, overlap)	69.9
4′	–	147.9	5″	3.41 (1H, m, overlap)	76.8
5′	7.16 (1H, d, 8.4)	116.6	6″	3.87 (1H, m, overlap) 3.71 (1H, m, overlap)	61.0

d, doublet; dd, doublet doublet; H, hydrogen; m, multiplet.

### BG measurement

The BG was measured in a drop of blood taken from the tail vein using a glucose test strip (GA‐3 type; Sinocare Inc., Shenzhen, China). Then the test strip was inserted into the BG meter (GA‐3 type; Sinocare Inc.), and the BG data shown in the panel were recorded.

### Kidney index

During the treatment, the rats in each group were weighed with an analytical balance (JJ224BC type; G&G Measurement Plant, Changshu, China) every week. The data were recorded. After the rats were sacrificed, the kidneys on both sides were quickly cut out. The residual blood was removed by lavage with precooled normal saline. After removing the excess fluid on the left and right kidneys, we chose an analytical balance to weigh the masses of both kidneys and recorded the data. Kidney index (KI) is a good indicator of renal function. The KI [KI = weight of left and right kidneys/(rat weight × 2)] based on the weight of the rat during the fourth week of treatment was calculated.

### ELISA

Blood was centrifuged at 1000 ***g*** for 15 min at 2–8 °C. The supernatant was collected. After that, the detection of inflammatory cytokines TNF‐α, IL1β and IL‐6 in each group of rats was performed using ELISA kits (CSB‐E11987r, CSB‐E08055r, CSB‐E04640r; CusaBio, Wuhan, China), respectively. The experimental instructions were followed strictly. A microplate reader was used to monitor the absorbance (*A*) values of each group at 450 nm wavelength (*A*_450_ _nm_). The content of blood urea nitrogen (BUN), creatinine (CRE), GSH, MDA, SOD and albumin (ALB) was detected by the kits (#C013‐2‐1, #C011‐2‐1, #A028‐2‐1, #A006‐2‐1, #A003‐1 and #A001‐3; Nanjing Jiancheng Bioengineering Institute, Nanjing, China), respectively. The operation was performed according to the manufacturer's instructions. The microplate reader (MB‐530; HEALES, Shenzhen, China) was used to measure the *A* values of each group at the wavelengths of 640 nm (BUN), 546 nm (CRE), 628 nm/630 nm (GSH), 405 nm (MDA), 532 nm (SOD) and 450 nm (ALB). The urinary ALB to CRE ratios (UACRs) were calculated.

### Western blot

First, the right kidneys of each group were taken out from −80 °C. We cut the appropriate amount of right kidney (about 0.025 g) or resuspended cells from each group, adding 250 µL radioimmunoprecipitation lysis buffer (P0013B; Beyotime, Shanghai, China) to lyse the samples. The cell supernatant was obtained by centrifugation. The instructions of the BCA protein quantification kit were followed to determine the protein concentration. Next, the same quantity of protein was taken to load it on the Bolt Bis‐Tris gel. After electrophoresis, the protein was transferred to the membrane. The membrane was blocked in 5% skimmed milk in TBST. We added primary antibodies to the samples, including Nrf2 (16396‐1‐AP, 1 : 1000; Proteintech, Chicago, IL, USA), HO‐1 (10701‐1‐AP, 1 : 3000; Proteintech) or internal reference β‐tubulin (10094‐1‐AP, 1 : 3000; Proteintech). They were incubated at 4 °C for a night. HRP‐Goat anti‐Rabbit IgG (SA00001‐2, 1 : 6000; Proteintech) was the secondary antibody. The sample was exposed to ECL development (Chemiscope 6100; CLINX, Shanghai, China). The integrated density of protein bands was determined by the quantity one 4.6.2 software (Bio‐Rad, Hercules, CA, USA) and corrected by subtracting the measured integrated density with the background integrated density. The proteins expression was calculated by the ratio of the integrated density of each target protein to the integrated density of the total β‐tubulin. Moreover, cell samples were tested: GSK‐3β (ab32391, 1 : 5000; Abcam, Cambridge, MA, USA) and p‐GSK‐3β (sc‐373800, 1 : 100; Santa Cruz Biotechnology, Santa Cruz, CA, USA). The processing steps and detection methods were the same as earlier.

### Quantitative real‐time PCR

The total RNA of kidney tissues and HRMCs in each group were isolated using TRIzol^®^ reagent (15596026; Thermo Fisher, Waltham, MA, USA). cDNAs were synthesized using mRNA reverse transcription kit (CW2569; CWBIO, Beijing, China). The PCR was performed using Ultra SYBR Mixture (CW2601; CWBIO). The fluorescent quantitative PCR system was Thermo Fisher (PIKOREAL96). The 2^−ΔΔCt^ method was applied to calculate the relative expression level. Actin and 5S were selected as internal reference mRNA and microRNA. The primer sequences (Shenggong, Shanghai, China) are listed in Table 2.

**Table 2:** The primer sequences used in this study. F, forward; R, reverse.

	Primer sequences
Actin	F: ACATCCGTAAAGACCTCTATGCC R: TACTCCTGCTTGCTGATCCAC
HO‐1	F: TCTGGAATGGAAGGAGATGC R: AGTTCTGGGGCTCTGTTGC
Nrf2	F: AGTGCAAGGCGGAGGTGA R: AGCCCGTTGGTGAACATAG

### Hematoxylin and eosin staining

After the right kidney tissues were taken from rats, they were fixed with 4% paraformaldehyde at room temperature for more than 24 h. After that, samples were dehydrated in gradient ethanol and embedded into a wax block. Each sample was cut into 2‐ to 3‐μm slices. Subsequently, slices were baked at 62 °C for 2–6 h, then dewaxed and rehydrated. The cytoplasm was stained with eosin to different degrees of red or pink, in sharp contrast with the blue nucleus stained with hematoxylin. Slices were observed under an optical microscope (BA210T; MOTIC, Xiamen, China).

### Immunohistochemistry

The expression of Nrf2 was detected by immunohistochemistry (IHC) in kidney tissues. Paraffin tissues were cut into 2‐ to 3‐μm slices and baked at 62 °C for 8 h. Slices were dewaxed and rehydrated. Then slices were heated to repair the antigen using 0.01 m citrate buffer (pH 6.0) (WELLBIO, Changsha, China) in a microwave oven (MM721NG1‐PS; MIDEA, Foshan, China) and blocked with 1% periodic acid (WELLBIO) at room temperature for 20 min. Samples were incubated with primary antibody Nrf2 (16396‐1‐AP, 1 : 100; Proteintech) overnight at 4 °C. PBS buffer (pH 7.4) was performed as a negative control instead of the primary antibody. The ready‐to‐use secondary antibody kit (PV9001; Zhongshan Jinqiao, Zhongshan, China) was chosen to incubate the samples for 30 min at 37 ℃. DAB (ZLI‐9018; Zhongshan Jinqiao) was used to develop color. The nucleus was stained with hematoxylin. We observed slices under an optical microscope and selected the location of the kidney cortex taking a 400‐fold field of view. Integral optical density (IOD) was the ratio of the cumulative optical density of the positive‐expressed site under the visual field to the sample area under visual field (for the 400‐fold field of view). image pro plus 6.0 image analysis software (Media Cybernetics, SilverSpring, MD, USA) was chosen for IOD analysis. The average optical density (positive area IOD under the field of view/tissue area under the field of view) was performed to indicate the relative expression of Nrf2.

### HRMCs culture

HRMCs (CP‐H067) were purchased from Wuhan PROCELL Life Technology Co., Ltd (Wuhan, China). HRMCs were added with 10% FBS medium, at 37 °C, 5% CO_2_ and 95% relative humidity for 2–3 days. After cells adhered to the well to 80% confluence, they were split into two for passage. HRMCs in the logarithmic growth phase were placed in Dulbecco’s modified Eagle’s medium (DMEM) or 30 mm glucose [hign glucose (HG)] DMEM for 24 h. Different concentrations of Isoeucommin A were added into DMEM, and the final concentration was adjusted to 0 μm (control group), 31.25 μm (low‐Isoeucommin A group), 61.3 μm (medium‐Isoeucommin A group) or 125 μm (high‐Isoeucommin A group). Isoeucommin A was performed to treat cells for 24 h continually.

### Isolation and culture RTECs of Sprague Dawley rat

The renal cortex separated from normal rat kidneys was cut into fragments, digested and applied to type II collagenase. The fragments of tissue were filtered to separate RTECs. Then RTECs were fostered under the same cultural conditions as HRMCs. Cells were divided into control group, model group (H_2_O_2_ treatment group), low‐Isoeucommin A (31.25 μm) group, medium‐Isoeucommin A (61.3 μm) group and high‐Isoeucommin A (125 μm) group. Both the model group and the administration group were added to DMEM containing 400 μmol·L^−1^ H_2_O_2_ and cultured in a 37 °C incubator for 2 h.

### MTT method to measure cell viability

Fifty milligrams of 3‐(4,5‐dimethylthiazol‐2‐yl)‐2,5‐diphenyl‐tetrazolium bromide(MTT) powder was diluted by 10 mL PBS to 5 mg·mL^−1^ test solution. It was filtered with 0.22‐µm filter membrane, aliquoted and stored at −20℃. Each group of RTECs was added to 100 µL of MTT detection solution. Cells were placed in a constant temperature shaker for 15 min at room temperature. The *A*_500_ _nm_ of the microplate reader was monitored. Then the cell viability was measured.

### Statistical analysis

All data were analyzed by graphpad prism 8.0 software (GraphPad Software, San Diego, CA, USA) and expressed as mean ± standard deviation. All experiments were repeated three times independently. Comparisons among multiple groups were conducted by one‐way ANOVA, followed by Tukey's *post hoc* test. *P* < 0.05 was considered statistically significant.

## Results

### Isoeucommin A attenuated the impaired renal function of DN rats

**Fig. 1:**
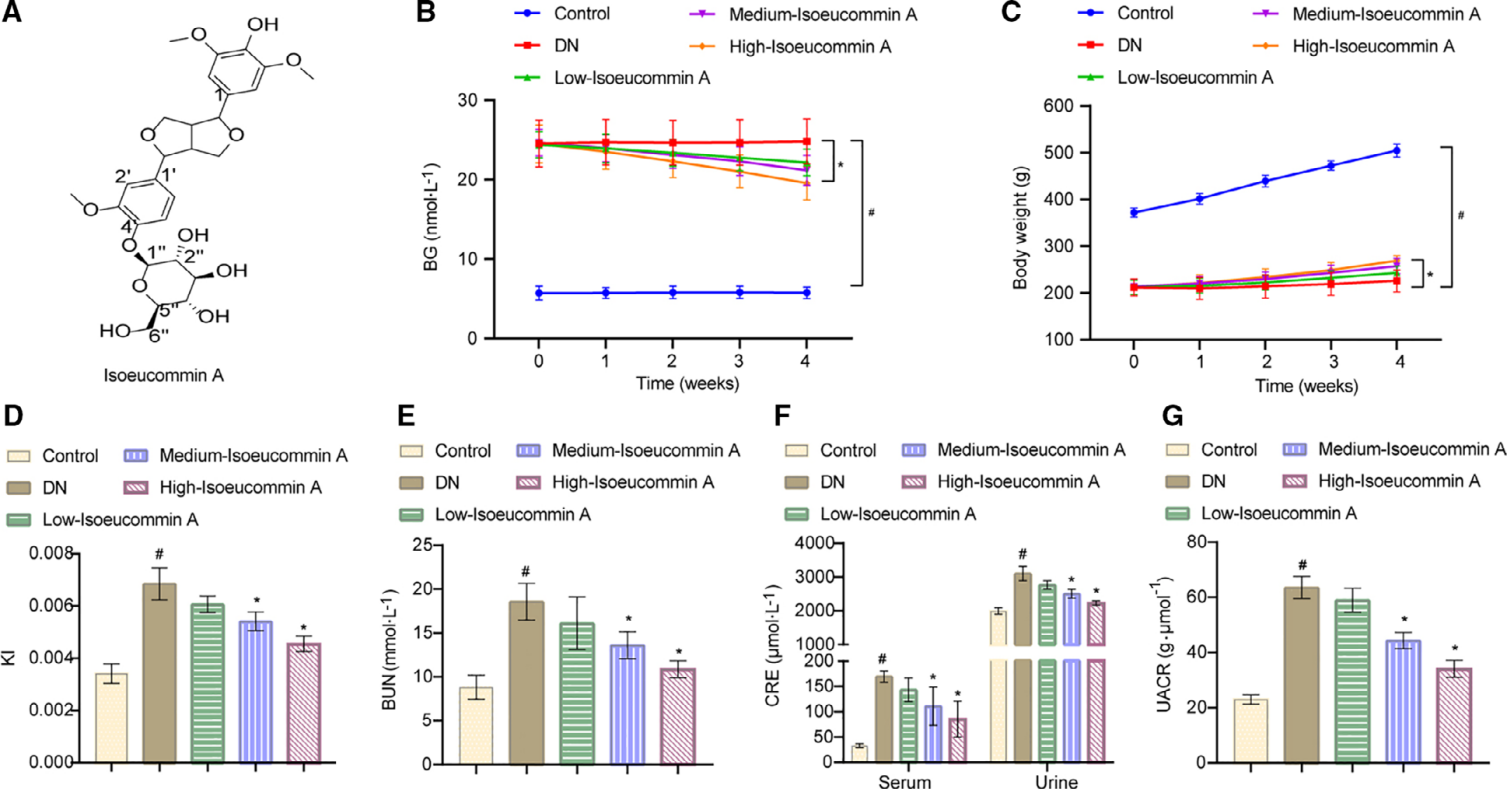
Isoeucommin A attenuated the impaired renal function of DN rats. (A) The structure of Isoeucommin A was obtained from the carbon NMR spectrum. (B) The BG status of each group was monitored weekly. (C) The body weight of rats in each group was monitored weekly. (D) The KI of rats in each group. (E) The level of BUN in the serum of rats in each group. (F) The levels of CRE in the serum and urine of rats in each group. (G) The UACR level of rats in each group. *n* = 3. All data were expressed as mean ± standard deviation. Comparisons among multiple groups were conducted by one‐way ANOVA, followed by Tukey's *post hoc* test. ^#^ *P* < 0.05 vs. control group; *P < 0.05 vs. DN group.

As shown in Fig. S1, we obtained the base peak chromatogram and extracted ion chromatogram and mass spectrum of EUO (Fig. S1A,C,E) and Isoeucommin A (Fig. S1B,D,F). By analyzing the retention time and mass spectrum information of the chemical components in EUO and comparing them with reference substances and databases, we concluded that Isoeucommin A was a component of EUO (Fig. S1A–D). Figure 1E,F showed that the molecular formula of C_27_H_34_O_12_ was determined by HRESIMS at *m/z* 573.1951 [M+Na] (calculated as 573.1942). The structure of Isoeucommin A was obtained according to the carbon NMR database (Fig. 1A). The earlier data were consistent with previous reports [19]. Thus, the compound was identified as Isoeucommin A. Compared with the control group, the BG of rats in the DN group was elevated markedly (^#^ *P* < 0.05). In addition, the BG level of rats in the Isoeucommin A groups was reduced compared with the DN group. However, only the high‐Isoeucommin A group had a statistical significance (*P < 0.05; Fig. 1B). DN treatment resulted in significant weight loss in rats (^#^ *P* < 0.05). In addition, the weekly weight gain of DN rats was extremely low, but Isoeucommin A treatment could augment the weight gain amplitude of DN rats in a dose‐dependent manner. The increase of the high‐Isoeucommin A group was more obvious, with statistical significance (*P < 0.05; Fig. 1C). Compared with the control group, the KI of rats in the DN group was significantly improved (^#^ *P* < 0.05). Under the Isoeucommin A treatment condition, the KI of DN rats was reduced, especially in the medium‐ and high‐Isoeucommin A groups (*P < 0.05; Fig. 1D). As the Isoeucommin A concentration increased, the levels of BUN, CRE (both serum and urine) and UACR in the administration groups were decreased sequentially. Compared with the DN group, all the indexes in the medium‐ and high‐Isoeucommin A groups were significantly reduced (*P < 0.05; Fig. 1E–G). These data suggested that Isoeucommin A treatment had time‐ and concentration‐dependent therapeutic effects on the impaired renal function of DN rats.

### Isoeucommin A reduced kidney tissue damage in DN rats

**Fig. 2:**
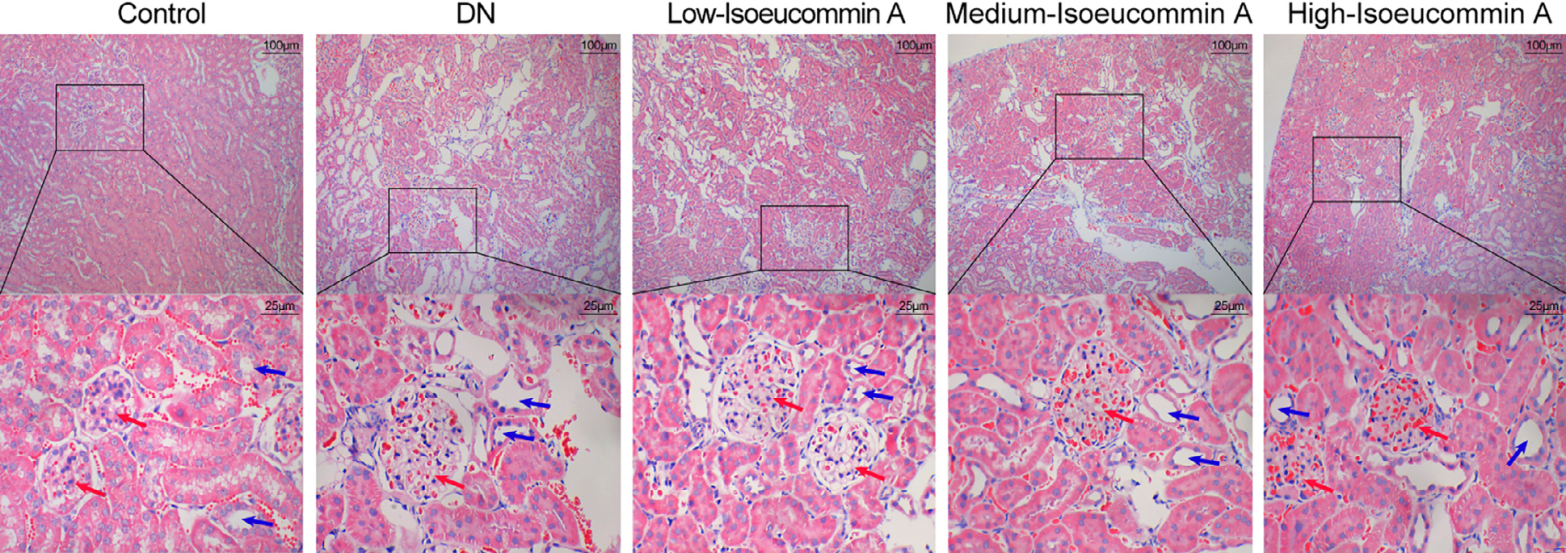
Isoeucommin A treatment could alleviate kidney tissue damage in DN rats. Hematoxylin and eosin staining was observed in the pathological changes of kidney tissue in each group. The magnification of upper images is 100‐fold (scale bar: 100 μm) and the local magnification (bottom) is 400‐fold (scale bar: 25 μm). The blue area represents nuclei. The pink area represents cytoplasm and intercellular plasm. The red arrows showed the position of the glomeruli, and the blue arrows showed the position of renal tubules.

In the control group, the morphology of the glomeruli and renal tubules was normal. As shown in Fig. 2, the red arrows showed the position of glomeruli, and the blue arrows showed the position of renal tubules. In contrast, substantial inflammatory cell infiltration was seen in the DN group. The glomerulus volume was enlarged. The renal tubules were slightly or moderately dilated. The RTECs were vacuolated. Nevertheless, the infiltration of inflammatory cells was significantly reduced after Isoeucommin A treatment. The glomerulus volume was relatively small, and dilation of renal tubules was prevented, especially the high‐Isoeucommin A group was close to the normal group. The vacuolation of RTECs was not obvious. The images showed that Isoeucommin provided some relief to damaged renal tissue in DN rats.

### Isoeucommin A relieved inflammation and oxidative stress in DN rats

**Fig. 3:**
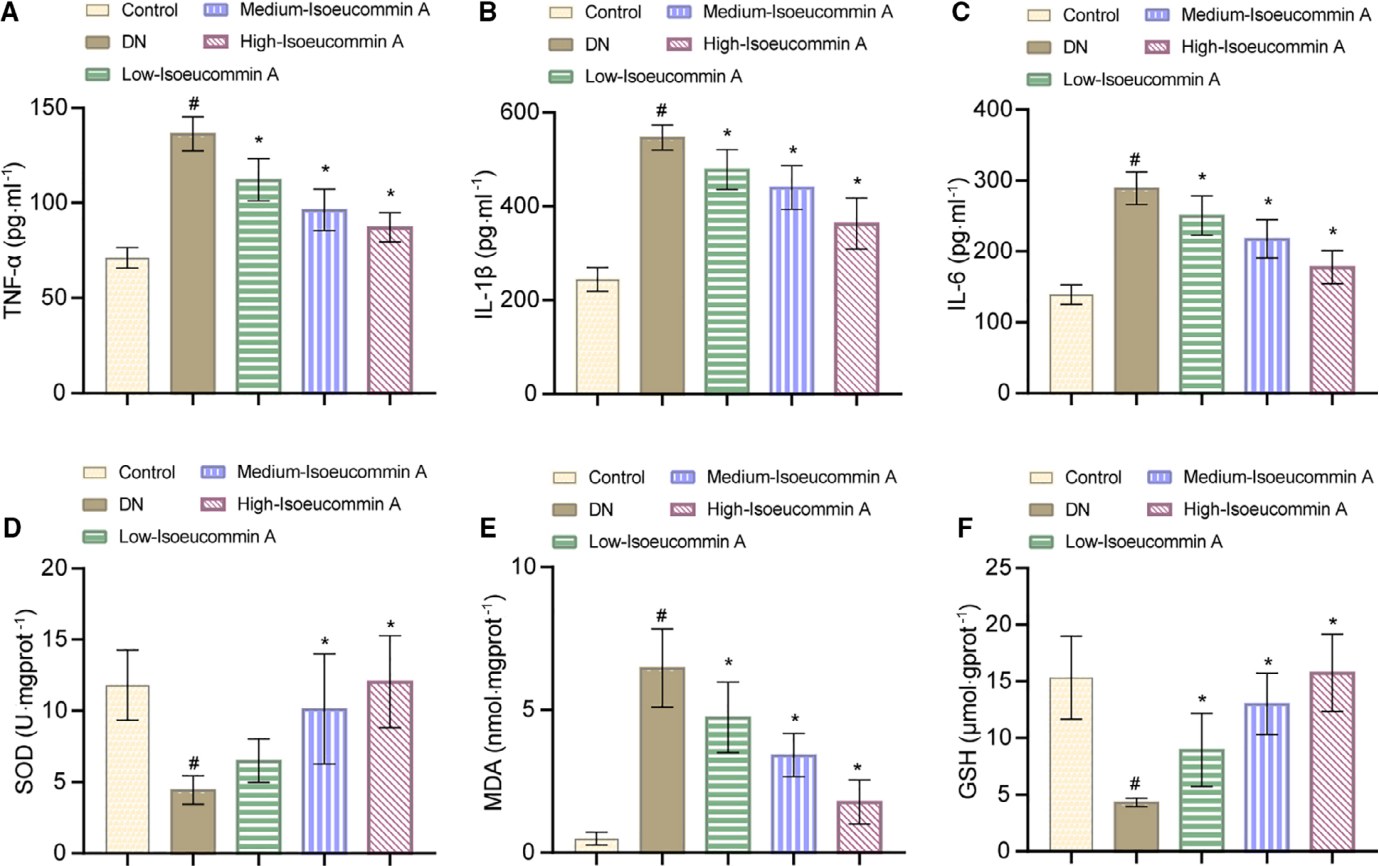
Isoeucommin A relieved inflammation and oxidative stress in DN rats. (A–C) The levels of TNF‐α, IL‐1β and IL‐6 in the serum of rats in each group. (D–F) The levels of SOD, MDA and GSH in the kidney tissues of rats in each group. *n* = 3. All data were expressed as mean ± standard deviation. Comparisons among multiple groups were conducted by one‐way ANOVA, followed by Tukey's *post hoc* test. ^#^ *P* < 0.05 vs. control group; *P < 0.05 vs. DN group.

Compared with the control group, the levels of TNF‐α, IL‐1β, IL‐6 (in serum) and MDA (in kidney tissues) in the DN group were increased (^#^ *P* < 0.05). Isoeucommin A treatment significantly down‐regulated the production of these inflammatory factors and MDA in DN rats as shown in Fig. 3A–C,E (*P < 0.05). The content of SOD and GSH was decreased markedly in the DN group compared with the control group (^#^ *P* < 0.05), although SOD and GSH levels of the DN rats have improved notably after Isoeucommin A treatment (Fig. 3D,F). In addition, as the concentration of Isoeucommin A increases, its effect becomes more obvious. Altogether, Isoeucommin A treatment had concentration‐dependent therapeutic effects on the alleviation of inflammation and oxidative stress in DN rats.

### Isoeucommin A attenuated kidney damage through the Nrf2/HO‐1 pathway in DN rats

**Fig. 4:**
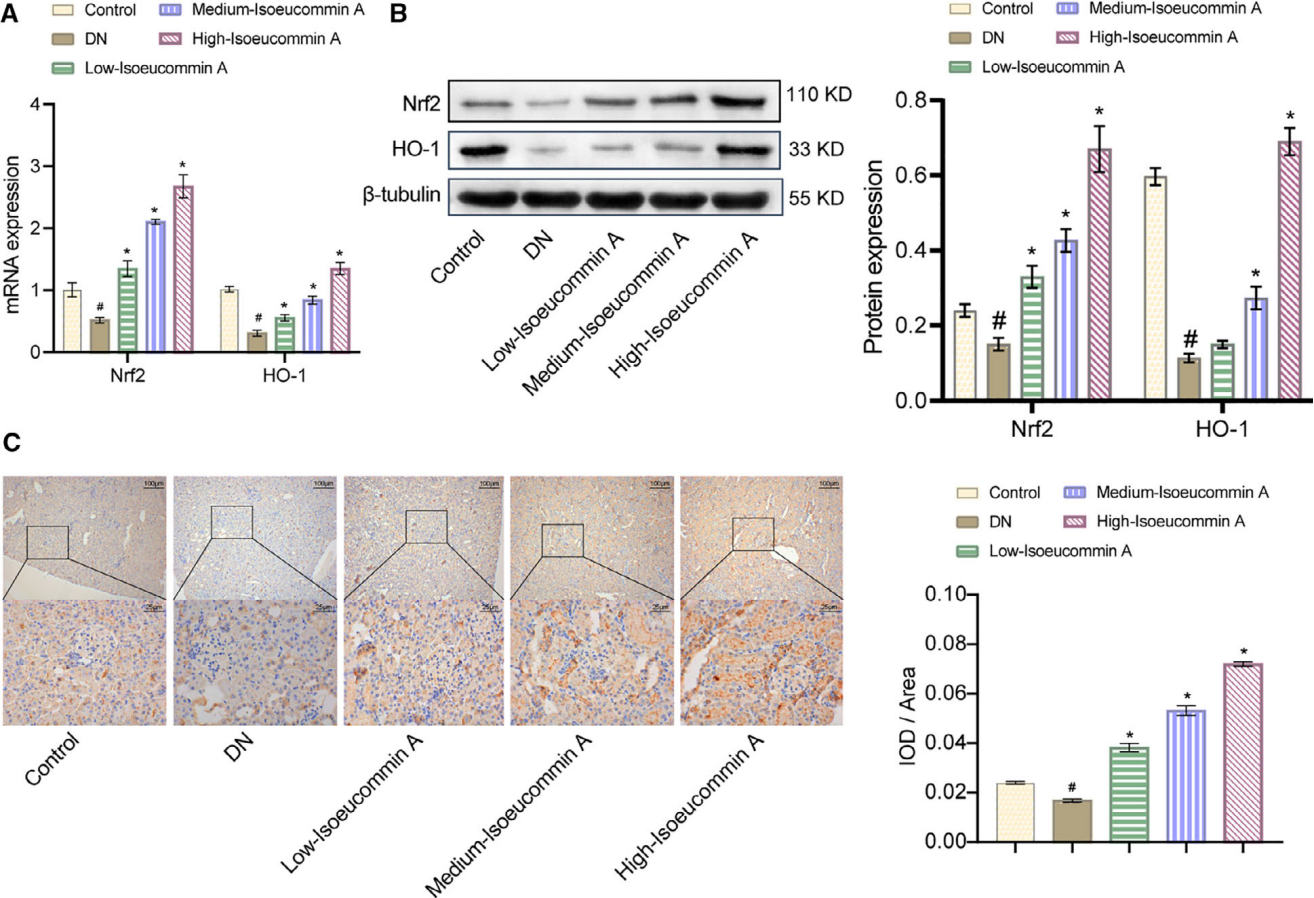
Isoeucommin A regulated the Nrf2/HO‐1 pathway to relieve kidney damage in DN rats. (A) The expression of Nrf2 and HO‐1 in the kidney tissues of rats in each group was detected by quantitative real‐time PCR at the molecular level. (B) Western blot was used to monitor the protein expression of Nrf2 and HO‐1 in the kidney tissues of rats in each group. (C) IHC was performed to measure the distribution of Nrf2 in the kidney tissues of rats in each group [original magnification ×100, scale bar: 100 μm (top); original magnification ×400, scale bar: 25 μm (bottom)]. *n* = 3. All data were expressed as mean ± standard deviation. Comparisons among multiple groups were conducted by one‐way ANOVA, followed by Tukey's *post hoc* test. ^#^ *P* < 0.05 vs. control group; *P < 0.05 vs. DN group.

To identify the effect of Isoeucommin A on the renal Nrf2/HO‐1 pathway in DN rats, we performed experimental validation at the molecular, protein and pathological levels, respectively. Quantitative real‐time PCR experiment results showed that the expression of HO‐1 and Nrf2 in the DN group was significantly lower than that in the control group (^#^ *P* < 0.05), while the expression in the Isoeucommin A group was significantly higher than that of the DN group (*P < 0.05). This effect was enhanced by the increased concentration of Isoeucommin A (Fig. 4A). The western blot and IHC results were consistent with the earlier results, as displayed in Fig. 4B,C. These results showed that Isoeucommin A could activate the Nrf2/HO‐1 pathway of DN rat kidney tissues.

### Isoeucommin A activated the Nrf2/HO‐1 signaling pathway in high‐glucose‐stimulated HRMCs

**Fig. 5:**
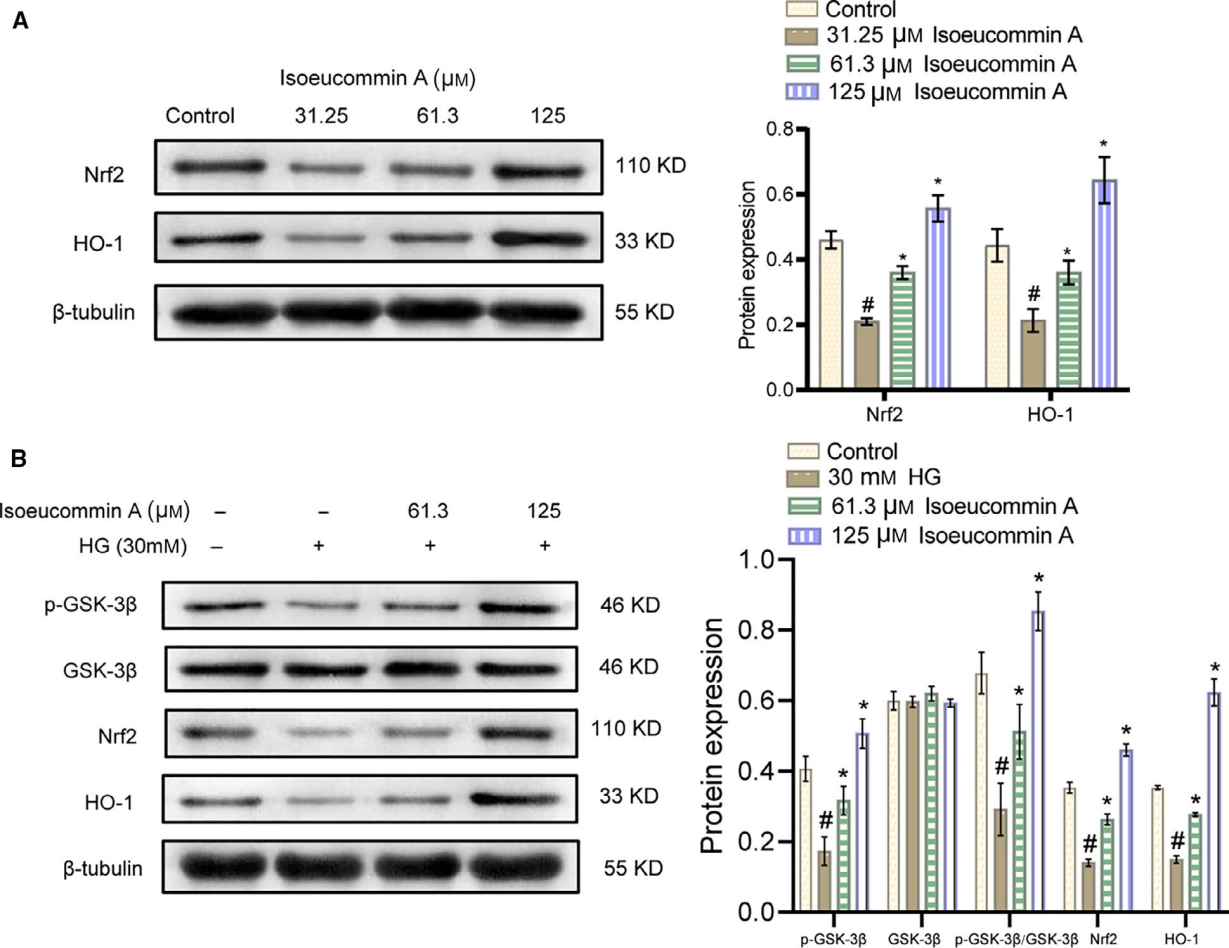
Isoeucommin A effectively activated the Nrf2/HO‐1 signaling pathway in normal and high‐glucose‐stimulated HRMCs. (A) Western blot was used to monitor the expression levels of Nrf2 and HO‐1 proteins in normal HRMCs. ^#^ *P* < 0.05 vs. control group; *P < 0.05 vs. 3.25 μm Isoeucommin A group. (B) Western blot was performed to test the expression levels of p‐GSK‐3β, GSK‐3β, Nrf2 and HO‐1 proteins in HRMCs stimulated by high glucose. *n* = 3. All data were expressed as mean ± standard deviation. Comparisons among multiple groups were conducted by one‐way ANOVA, followed by Tukey's *post hoc* test. ^#^ *P* < 0.05 vs. control group; *P < 0.05 vs. HG group.

Next, we further verified whether Isoeucommin A could interfere with the Nrf2/HO‐1 signaling pathway in high‐glucose‐stimulated HRMCs. As the western blot image displayed, compared with the control group, the expressions of Nrf2 and HO‐1 were elevated as the concentration of Isoeucommin A improved (Fig. 5A). These results indicated that Isoeucommin A with high content could enhance Nrf2/HO‐1 pathway activity in normal HRMCs. Yet, Nrf2 expression in low Isoeucommin A was almost unchanged, so we abandoned this concentration for subsequent studies. The high‐glucose treatment reduced the expression of Nrf2 and HO‐1 in HRMCs. With the addition of Isoeucommin A, the expression of phosphorylation of GSK‐3β (p‐GSK‐3β), p‐GSK‐3β/GSK‐3β, Nrf2 and HO‐1 was increased accordingly in HRMCs stimulated by high glucose (Fig. 5B). The p‐GSK‐3β was significantly elevated after Isoeucommin A treatment, which decreased the protein stability of GSK‐3β and reduced its inhibitory effect of Nrf2. Hence Isoeucommin A could also activate the Nrf2/HO‐1 signaling pathway in high‐glucose‐stimulated HRMCs.

### Isoeucommin A enhanced the antioxidation in high‐glucose‐stimulated HRMCs

**Fig. 6:**
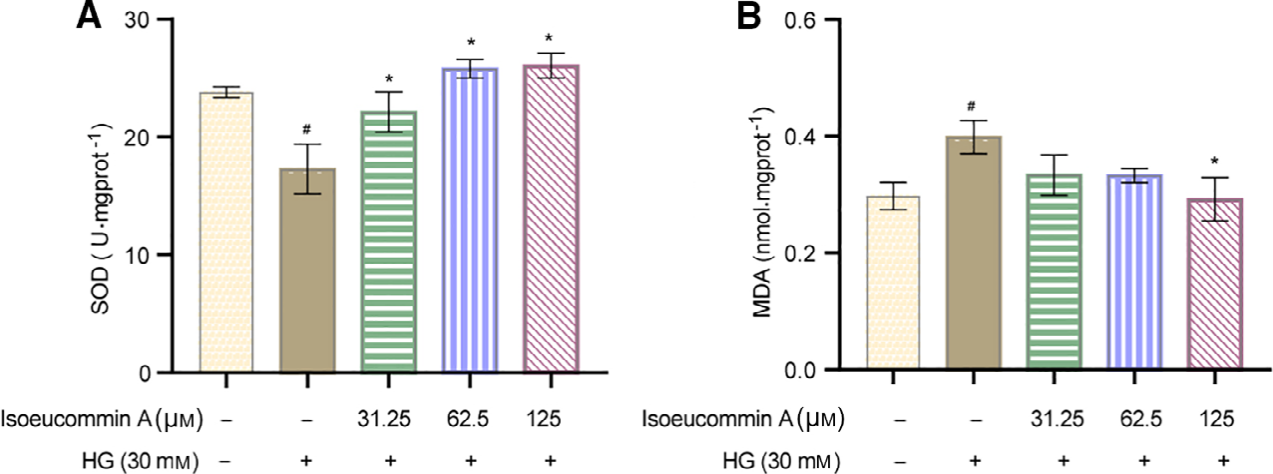
Isoeucommin A enhanced the antioxidation in HRMCs stimulated by high glucose. (A) The SOD level in the cells of each group. (B) The kit detected the MDA expression level of each group of cells. *n* = 3. All data were expressed as mean ± standard deviation. Comparisons among multiple groups were conducted by one‐way ANOVA, followed by Tukey's *post hoc* test. ^#^ *P* < 0.05 vs. control group; *P < 0.05 vs. HG group.

Next, we detected the expression levels of antioxidant SOD and oxidative stress factor MDA in each group of HRMCs. We found that the SOD level in the HG group was reduced (^#^ *P* < 0.05) compared with the control group, whereas the MDA level was increased (^#^ *P* < 0.05). In addition, the content of SOD in the Isoeucommin A+HG groups was increased (*P < 0.05) compared with the HG group, but MDA content was decreased (*P < 0.05; Fig. 6A,B). The results showed that Isoeucommin A could reduce the oxidation induced by high glucose and thus protect HRMCs.

### Isoeucommin A protected H_2_O_2_‐stimulated RTECs from oxidative stress

**Fig. 7:**
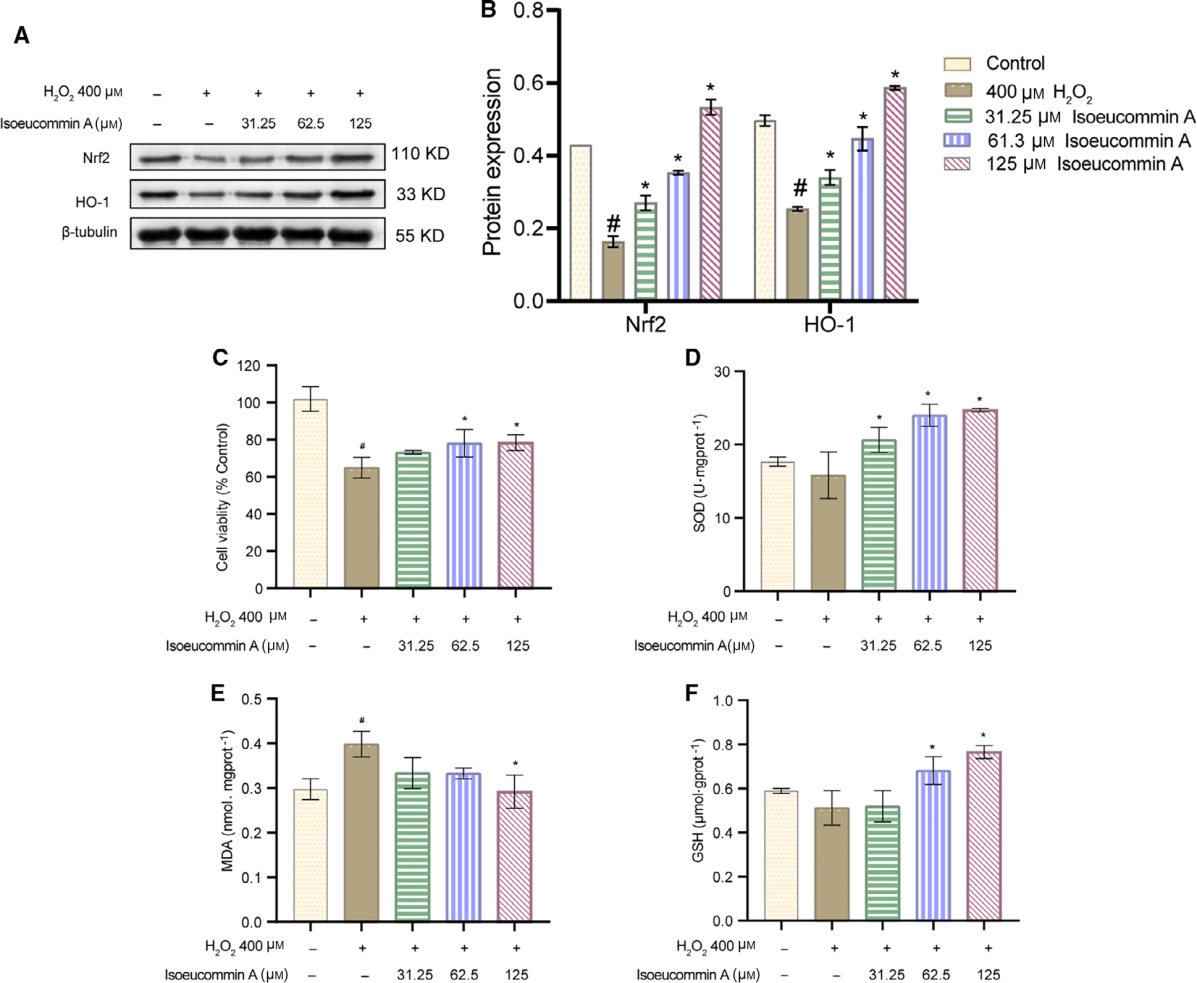
Isoeucommin A could effectively inhibit the oxidation signal pathway in RTECs of Sprague Dawley rats injured by H_2_O_2_. (A, B) Western blot was used to detect the expression levels of Nrf2 and HO‐1 protein in RTCEs in each group. (C) The MTT method was performed to test the viability of RTCEs in each group. (D–F) The biochemical kit monitored the enzyme activity levels of SOD, MDA and GSH in RTCEs in each group. *n* = 3. All data were expressed as mean ± standard deviation. Comparisons among multiple groups were conducted by one‐way ANOVA, followed by Tukey's *post hoc* test. ^#^ *P* < 0.05 vs. control group; *P < 0.05 vs. 400 μm H_2_O_2_ group.

To explore the effect of Isoeucommin A on the RTECs injured by H_2_O_2_, we first examined the expression of Nrf2/HO‐1 signal pathway‐related proteins. As shown in Fig. 7A,B, with the stimulation of H_2_O_2_ in RTECs, the expressions of HO‐1 and Nrf2 were down‐regulated. When the Isoeucommin A was added into H_2_O_2_‐stimulated RTECs, the expression of both was improved robustly in a concentration‐dependent manner. As shown in Fig. 7C, in contrast with the control group, H_2_O_2_ treatment reduced the cell viability with significant differences (^#^ *P* < 0.05). The cell viability was advanced with the addition of Isoeucommin A, in particular, the medium‐ and high‐concentration groups (*P < 0.05). The compound Isoeucommin A might inhibit the killing effect of H_2_O_2_ on RTECs to a certain extent. Then the role of Isoeucommin A on oxidative stress of H_2_O_2_‐stimulated RTECs was analyzed. Through determining the content of SOD, MDA and GSH, the data in Fig. 7D–F indicated that H_2_O_2_ made SOD and GSH decline slightly, but MDA ascended apparently. After adding Isoeucommin A, the situation of three enzymes was reversed in H_2_O_2_‐stimulated RTECs. The levels of SOD and GSH both increased gradually accompanied by the increasing Isoeucommin A. Instead, MDA was diminished by Isoeucommin A, and the effect was most obvious in the high‐concentration group (*P < 0.05). The earlier experimental results illustrated that Isoeucommin A effectively activated the Nrf2/HO‐1 signal pathway to counteract the oxidative stress induced by H_2_O_2_ in RTECs.

## Discussion

In this study, Isoeucommin A was purified from EUO bark. HRESIMS analyzed the mass spectrum of Isoeucommin A, a lignan compound isolated from EUO, and the molecular formula was C_27_H_34_O_12_ at *m/z* 573.1951 [M+Na] (calculated as 573.1942). NMR spectroscopy is the approach for determining the structures of biological macromolecules [20]. Moreover, NMR is recognized as a critical platform for metabolomics in drug discovery [21]. In this study, the structure of Isoeucommin A was detected by NMR, and the data were obtained. These data were consistent with previous reports [19], so the compound was identified as Isoeucommin A. After a series of experimental analyses, we found that Isoeucommin A may have protective and therapeutic effects on DN. As we all know, BUN, SCR and UACR are the main indicators of DN [22]. Previous studies have found that the body weight of DN rats was often accompanied by the loss of weight and the increase of BG continuously [23]. Our results showed that compared with the DN model group, the body weight, BG, BUN, CRE and UACR in the Isoeucommin A treatment group were reduced in a time‐ and concentration‐dependent manner. Furthermore, the HRMCs in Isoeucommin A treatment groups were dead. Therefore, in this study, we speculated the Isoeucommin A plays an important protective and therapeutic effect on DN.

Inflammation promotes DN by leading to tubular fibrosis and glomerulosclerosis [24]. Al Hroob *et al*. [25] found that feeding STZ‐DN rats with *Zingiber officinale* extract can affect the expression of TNF‐α, IL‐1 β, IL‐6 and MDA (or SOD, GSH and CAT in rats) to achieve the therapeutic effect of DN. Oxidative stress is an important factor in the occurrence and development of DN [26]. TNF‐α, IL‐1β and IL‐6 are common inflammatory factors. MDA is the end product of the peroxidation of lipids by free radicals. The higher the level of MDA *in vivo*, the more serious is the oxidative stress reaction of the organism [27]. SOD and GSH are two kinds of important antioxidant markers. SOD catalyzes the conversion of superoxide to oxygen and hydrogen peroxide. GSH scavenges oxidants as part of the oxidative stress response within cells [28]. The imbalance in the production of reactive oxygen species and antioxidants is believed to be related to renal failure caused by diabetes [29]. Although the antioxidant enzymes are induced to protect cells and tissues from damage [30], more and more animal experiments and clinical studies have shown that diabetes may interfere with the antioxidant defense system by regulating the activity of antioxidant enzymes [31]. Stimulation of these antioxidants is an important therapeutic strategy for diseases characterized by increased oxidative stress [32]. We found that Isoeucommin A has a similar effect. In our study, the levels of the inflammatory factors TNF‐α, IL‐1β and IL‐6 in the treatment group were decreased and MDA level was decreased, whereas antioxidants SOD and GSH levels were increased. These results were consistent with previous studies [33], which showed that Isoeucommin A could protect and treat DN through the inhibition of oxidative stress and inflammation.

The Nrf2/HO‐1 signaling pathway can regulate the expression of various antioxidant factors [14]. Liu *et al*. [34] found that lignans extracted from EUO can activate Nrf2/ARE signal and promote Nrf2/HO‐1 signal transduction, to protect endothelial cells and prevent diabetic vascular complications. Previous studies found that hypothermia could prevent hippocampal oxidative stress and apoptosis via the GSK‐3β/Nrf2/HO‐1 signaling pathway in a rat model of cardiac arrest‐induced brain damage [35]. In addition, activation of the Nrf2/HO‐1 pathway by GSK‐3β inhibition attenuates renal ischemia/reperfusion injury in diabetic rats [36]. According to our study, Isoeucommin A can inhibit oxidative stress by regulating the Nrf2/HO‐1 signaling pathway in rats, thus protecting and treating DN rats. The phosphorylation level of GSK‐3β was significantly elevated after Isoeucommin A treatment, which decreased the protein stability of GSK‐3β and reduced its inhibitory effect of Nrf2. At the cellular level, Isoeucommin A can also enhance the antioxidant effect of HRMCs induced by high glucose by regulating Nrf2/HO‐1. Isoeucommin A could effectively up‐regulate Nrf2/HO‐1 in RTECs of Sprague Dawley rats injured by H_2_O_2_.

HRMCs are essential for maintaining the human glomerular capillary structure and regulating its function [37]. DN is a major long‐term microvascular complication of uncontrolled hyperglycemia [38]. In addition, hyperglycemia is the main driving force of DN progressing to end‐stage renal disease [39]. A previous study revealed that *Allium tuberosum* could alleviate DN by suppressing hyperglycemia‐induced oxidative stress and inflammation in high‐fat‐diet/STZ‐treated rats [40]. Therefore, exposure to high glucose in patients with DN will lead to the oxidative stress of HRMCs. To solve this problem, some studies have found that the purple corn extract rich in anthocyanins can treat DN under hyperglycemia [37]. *Allium tuberosum* could alleviate DN by suppressing hyperglycemia‐induced oxidative stress and inflammation [40]. High‐glucose‐stimulated HRMCs were used to verify the protective and therapeutic effects of Isoeucommin A on DN in our study. Isoeucommin A can effectively activate the Nrf2/HO‐1 signaling pathway in high‐glucose‐stimulated HRMCs. In this study, we used high glucose to stimulate HRMCs to induce oxidative stress. It was found that the content of MDA was increased, whereas the content of SOD and GSH were decreased. Isoeucommin A could increase the levels of SOD and GSH, inhibit the expression of MDA and reduce the inhibition of Nrf2, thus promoting the expression of HO‐1.

Due to the limitation of our experimental conditions, that is, time and funding constraints, our animal experimental sample amount is relatively small. We have not done clinical experiments. For the safety of the drug, we did not perform a complete test. What is more, our research could hardly show that Nrf2 is a mediator of the cytoprotective effect of Isoeucommin A. In future work, we will make up these deficiencies and use Nrf2 knockdown or knockout rat experiments to verify that Nrf2 is a mediator of the cytoprotective effect of Isoeucommin A. Furthermore, we will add clinical trials to confirm the therapeutic effect of Isoeucommin A in DN and determine the safety and toxicity of Isoeucommin A.

## Conclusions

We found that Isoeucommin A could activate the Nrf2/HO‐1 pathway and effectively alleviate inflammatory and oxidative stress of injured kidneys in DN through *in vitro* and *in vivo* experiments. Isoeucommin A would be a potential drug for the treatment of DN.

## Conflict of interest

The authors declare no conflict of interest.

## Author contributions

QH performed the experiment and analyzed the data. QL performed the experiment and edited the manuscript. D‐SO guided the experiment and reviewed the manuscript.

## Supporting information

Additional supporting information may be found online in the Supporting Information section at the end of the article.’

**Fig. S1**. Comparison of EUO and Isoeucommin A. (A‐B) The base peak chromatogram (BPC) of EUO and Isoeucommin A. (C‐D) The extracted ion chromatogram (EIC) of EUO and Isoeucommin A. (E‐F) The mass spectrum of EUO and Isoeucommin A. Diabetic nephropathy (DN) is a common complication in patients with diabetes. Here we investigated Isoeucommin A treatment *in vitro*, as well as in a rat model of DN. We observed a dose dependent increase in the expression levels of SOD, GSH, Nrf2, HO‐1, and p‐GSK‐3β/GSK‐3β in DN rats upon treatment. On the other hand, TNF‐α, IL‐1β, IL‐6, and MDA levels decreased significantly. Isoeucommin A protected H_2_O_2_‐stimulated renal tubular epithelial cells (RTECs) from oxidative stress and activated the Nrf2/HO‐1 signaling pathway in high glucose‐stimulated human renal mesangial cells (HRMCs).

## Data Availability

The datasets used and/or analyzed during this study are available from the corresponding author on reasonable request.
