# Supplementary figures and images for: Isoeucommin A attenuates kidney injury in diabetic nephropathy through the Nrf2/HO‐1 pathway
Source: FEBS Open Bio. 2021 Jul 24;11(8):2350–63. doi: 10.1002/2211-5463.13251 (PMC8329780; doi:10.1002/2211-5463.13251)

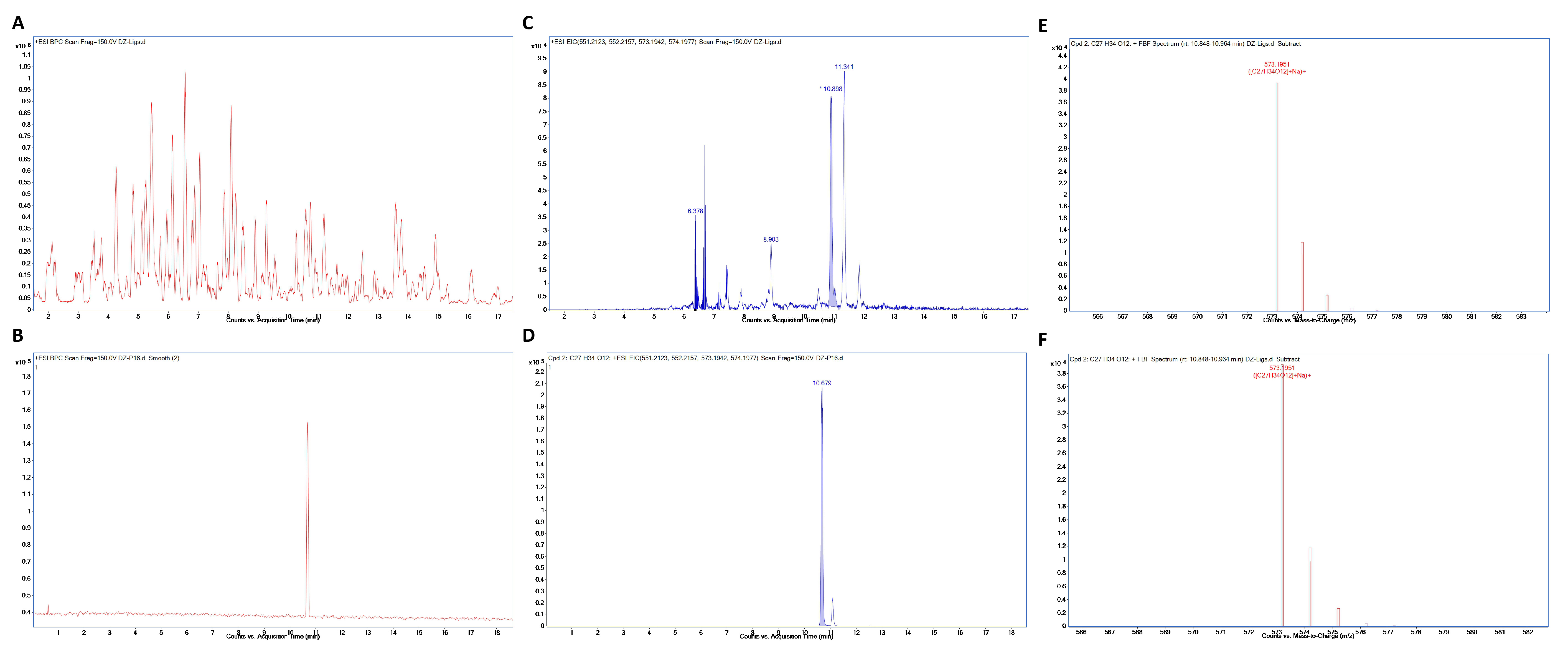

Supplement: Supplementary file 1 — Fig. S1. Comparison of EUO and Isoeucommin A. (A‐B) The base peak chromatogram (BPC) of EUO and Isoeucommin A. (C‐D) The extracted ion chromatogram (EIC) of EUO and Isoeucommin A. (E‐F) The mass spectrum of EUO and Isoeucommin A. Diabetic nephropathy (DN) is a common complication in patients with diabetes. Here we investigated Isoeucommin A treatment in vitro, as well as in a rat model of DN. We observed a dose dependent increase in the expression levels of SOD, GSH, Nrf2, HO‐1, and p‐GSK‐3β/GSK‐3β in DN rats upon treatment. On the other hand, TNF‐α, IL‐1β, IL‐6, and MDA levels decreased significantly. Isoeucommin A protected H2O2‐stimulated renal tubular epithelial cells (RTECs) from oxidative stress and activated the Nrf2/HO‐1 signaling pathway in high glucose‐stimulated human renal mesangial cells (HRMCs). [file FEB4-11-2350-s001.jpg]
